# The impact of second-hand smoke on nitrogen oxides concentrations in a small interior

**DOI:** 10.1038/s41598-021-90994-x

**Published:** 2021-06-03

**Authors:** Markus Braun, Doris Klingelhöfer, Ruth Müller, David A. Groneberg

**Affiliations:** 1 Institute of Occupational Medicine, Social Medicine and Environmental Medicine, Goethe University Frankfurt, Theodor-Stern-Kai 7, 60590 Frankfurt am Main, Germany; 2 Medical Entomology, Department of Biomedical Sciences, Institute of Tropical Medicine, Nationalestraat 155, 2000 Antwerp, Belgium

**Keywords:** Environmental sciences, Risk factors

## Abstract

Nitrogen oxides (NO_x_), especially nitrogen dioxide (NO_2_), are among the most hazardous forms of air pollution. Tobacco smoke is a main indoor source of NO_x_, but little information is available about their concentrations in second-hand smoke (SHS), particularly in small indoors. This study presents data of NO_x_ and its main components nitric oxide (NO) and NO_2_ in SHS emitted by ten different cigarette brands measured in a closed test chamber with a volume of 2.88 m^3^, similar to the volume of vehicle cabins. The results show substantial increases in NO_x_ concentrations when smoking only one cigarette. The NO_2_ mean concentrations ranged between 105 and 293 µg/m^3^, the NO_2_ peak concentrations between 126 and 357 µg/m^3^. That means the one-hour mean guideline of 200 µg/m^3^ for NO_2_ of the World Health Organization was exceeded up to 47%, respectively 79%. The measured NO_2_ values show positive correlations with the values for tar, nicotine, and carbon monoxide stated by the cigarette manufacturers. This study provides NO_2_ concentrations in SHS at health hazard levels. These data give rise to the necessity of health authorities’ measures to inform about and caution against NO_x_ exposure by smoking in indoor rooms.

## Introduction

Air pollution is potentially the greatest environmental risk factor for health worldwide. The *World Health Organization* (WHO) estimates 4.2 million premature deaths per year caused by ambient (outdoor) air pollution and 3.8 million premature deaths caused by indoor air pollution^1^. Since the sulfur dioxide concentrations in the atmosphere decreased in the last decades, the focus is set more and more on particulate matter (PM), ozone, and nitrogen dioxide (NO_2_) as the most hazardous air pollutions^2^.

Nitrogen oxides (NO_x_), mainly nitric oxide (NO), and, at a lower level, NO_2_, are gases formed by combustion processes at high temperatures^3^. The main anthropogenic outdoor sources are the combustion of fossil and green fuels in vehicles and power plants. Important indoor sources are oil-, gas-, kerosene-, coal-, and wood-burning ovens, heaters or open fireplaces, and tobacco smoke^4^. In the last years, NO_x_ emissions caused by road traffic and diesel vehicles, in particular, came into the focus of the public and policy. Emission limit values of engines were discussed and tightened. The aim was not to exceed the determined limit values of NO_2_ in ambient air too often, particularly in metropolitan areas. The air quality standards of the European Union for NO_2_ in ambient air are currently 200 µg/m^3^ (1 h mean, should not exceed more than 18 times each year) and an annual mean of 40 µg/m^3^ ^5^. Therewith, they followed the recommendations of the WHO air quality guidelines from 2005^6^. As indoor air pollution came more and more into the focus, the WHO published in 2010 guidelines for indoor air quality, whereby the NO_2_ limit values are consistent with the values for ambient air^4^.

NO_2_, in particular, is associated with a lot of adverse health effects on several organ systems^7^. Long-term exposure to NO_2_, besides PM, is an important risk factor for cardiopulmonary mortality^8^. Moreover, the NO_2_ concentration is positively associated with a variety of harmful effects, the mortality of respiratory and cardiovascular diseases, and lung cancer^9^.

NO is largely considered a toxic pollutant. The exposure can cause, among others, irritation to the skin, eyes, and respiratory system, but also unconsciousness and methemoglobinemia^10^. Albeit, NO may be hazardous to the health of humans not until relatively high doses^11^. For workplace atmospheres, the U.S. National Institute for Occupational Safety and Health gives an NO exposure limit value (8 h time-weighted-average) of 25 ppm (30 mg/m^3^)^10^. Under environmental conditions, NO will be quickly oxidized to NO_2_ by, e.g., ozone or oxygen. This oxidation process is, however, much slower under indoor conditions^4,12^.

Several studies investigated indoor air pollution, including NO_x_ respectively NO_2_ concentrations, caused by ovens (primarily gas stoves) or fireplaces for heating or cooking^13–19^. Some studies focused on tobacco smoke reporting a relatively lower influence on NO_x_ concentrations by burning tobacco products in normal-sized rooms or houses^20–22^. The impact of second-hand smoke (SHS) on NO_x_ burden in very small indoors like smoking cabins, telephone cells, or cabins of vehicles, for example, remains widely unclear. This study presents the results of the NO, NO_2_, and NO_x_ (NO + NO_2_) investigations in SHS of ten different cigarette brands with different strengths, additives, and origin in a 2.88 m^3^ measuring chamber. The measurements were realized as part of the Tobacco Smoke Particles and Indoor Air Quality (ToPIQ) studies^23^ at two PM investigations. The PM-related results of these studies are already described and published^24,25^.

## Material and methods

### Tobacco products

The concentrations of NO, NO_2_, and NO_x_ in SHS were measured of nine commercial cigarette brands (named cigarette A to I) and the 3R4F reference cigarette (Kentucky Tobacco Research and Development Center, University of Kentucky, USA)^26^. As the measurements took place during two PM investigations, the reference cigarettes (RC) were termed RC1^24^ and RC2^25^. The cigarette brands A, C, and E were bought at the International Airport of Dubai, United Arab Emirates (UAE), while the brands B, D, and F were from the International Airport of Frankfurt, Germany^25^. Additionally, three mentholated cigarette brands (G, H, I) were tested, purchased at the central station, Frankfurt, Germany^24^. The brand names of the cigarettes are given in Table 1. The cigarette brands differ in amounts of tar, nicotine, carbon monoxide (CO), and PM_10_ (Table 1). Further information on the cigarettes from Germany is available from the tobacco additives database of the Federal Ministry of Food and Agriculture of Germany^27^.

**Table 1:** Features of the tested cigarette brands.

Brand	Tested cigarettes (n)	Tar (mg)	Nicotine (mg)	Carbon monoxide (mg)	Particulate matter PM_10_ (µg/m^3^)	Format and attribute
Reference Cigarette 3R4F (RC1, RC2)	41 (RC1 = 21, RC2 = 20)	9.4	0.73	12	1147	KS Filter
Cigarette A Marlboro Gold UAE	20	6	0.5	7	1163	KS Filter
Cigarette B Marlboro Gold GER	20	6	0.5	7	874	KS Filter
Cigarette C Winston Red UAE	20	7	0.6	7	1016	KS Filter
Cigarette D Winston Classic GER	18	10	0.8	10	778	KS Filter
Cigarette E Parliament Platinum UAE	24	1	0.1	1	1099	KS Filter
Cigarette F Parliament Night Blue GER	24	10	0.8	10	1071	100 s Filter
Cigarette G Pall Mall Menthol Blast	15	10	0.8	10	1103	KS Filter, mentholated
Cigarette H Winston Menthol	22	10	0.8	10	1182	KS Filter, mentholated
Cigarette I Elixyr Menthol	21	10	0.8	10	1186	KS Filter, mentholated

Data on particulate matter (PM_10_) are measured mean concentrations. PM_10_ data of the reference cigarette RC1 and the cigarette brands G, H, and I were adjusted on the PM_10_ data of the reference cigarette RC2 by statistical data transformation. Information on the reference cigarette is taken from the manufacturer (Kentucky Tobacco Research and Development Center of the University of Kentucky). Information on the cigarettes A to I as specified by the manufacturers. *KS *King Size, *UAE *United Arab Emirates, *GER *Germany.

### Test chamber

All measurements took place in a test chamber with an internal volume of 2.88 m^3^. During the experiments, the vents for the supply and exhaust air were closed to minimize air exchange. The test chamber is placed in a laboratory room of the Institute of Occupational Medicine, Social Medicine and Environmental Medicine, Goethe University Frankfurt. The institute is located in an urban area but not near a traffic road. That avoided high NO_x_ concentrations in ambient air by road traffic (see baseline evaluation).

### Ambient nitrogen oxide monitor (NO_x_ monitor)

To ascertain the NO, NO_2_, and NO_x_ (NO + NO_2_) concentrations of SHS, the ambient nitrogen oxide monitor APNA-370 of HORIBA, Ltd. (Kyoto, Japan) was applied^28^. By using a cross-flow modulated semi-decompression chemiluminescence method, NO_2_ concentrations were internally calculated from those of NO and NO_x_. All measurement values were recorded in the unit ppm (parts per million) every three minutes. The not mixed sample air was collected at a point 40 cm above the burning tobacco product and 170 cm above the floor of the test chamber.

### Automatic environmental tobacco smoke emitter

SHS was generated in the closed test chamber by an Automatic Environmental Tobacco Smoke Emitter (AETSE), developed and constructed by Schimpf Ing. (Trondheim, Norway)^29^. This programmable microprocessor-controlled smoke pump imitated the smoker by moving a 200 ml glass syringe connected with the mouthpiece of the tobacco product via a polyamide tube. Moving the syringe led to puffing the tobacco product. Two valves controlled the air stream and pressed the mainstream smoke after each puff into the closed chamber. Between the puffs, the tobacco product smoldered and produced side-stream smoke. In doing so, no person was exposed to the generated SHS.

### Smoking protocol

The number (n) of investigated cigarettes of each brand varied between 15 and 41 (Table 1). According to the ToPIQ studies^23–25^, all cigarettes were smoked following a modified protocol. Puff volume was 40 ml, and the flow rate was 13 ml/s. After two ignition puffs, each cigarette was smoked in the combustion phase with seven puffs and a frequency of two puffs/min. Subsequently, the post-combustion phase followed after the extinguishing of the cigarette. After ten minutes in total, the chamber was ventilated with outdoor air by an industrial radial fan for at least five minutes to clean the air.

### Baseline evaluation

As the NO_x_ monitor detected data permanently, continuous measuring data of 61 h between the two measuring campaigns without SHS generation were chosen to determine the baseline values of NO, NO_2_, and NO_x_.

### Data processing

The NO_x_ monitor provided every three minutes measuring data. Therefore, in the 10-min combustion and post-combustion phase, three values of NO, NO_2_, and NO_x_, respectively, per investigated cigarette could be taken into account for the following data processing. The mean concentrations (C_mean_) of these three measuring values of NO, NO_2_, and NO_x_ were calculated. Additionally, the highest values of NO, NO_2_, and NO_x_ were considered as peak values (C_peak_) for each cigarette. For the statistical analysis, all C_mean_ and C_peak_ values were tested for outliers (Grubbs’ test). Sixteen outliers were detected and subsequently excluded from further statistical tests. All data were normally distributed. To compare the data of all investigated cigarettes, a one-way analysis of variance (ANOVA) including Tukey’s multiple comparison test was performed.

The associations of the C_mean_ values of NO, NO_2_, and NO_x_ with concentrations of tar, nicotine, CO, and PM_10_ were examined by using correlation analysis (Spearman) and linear regression. PM_10_ is classified by the US Environmental Protection Agency (EPA) as inhalable particles ≤ 10 µm and includes the fraction of the fine inhalable particles ≤ 2.5 µm (PM_2.5_)^30^. The measured PM_10_ C_mean_ values of RC1 were lower than those of RC2. Therefore, it was necessary to adjust the PM_10_ data of RC1 and the associated cigarette brands G, H, and I on the PM_10_ data of RC2 by statistical data transformation (Y = K*Y) using the factor K = 1.47.

Statistical analyses were performed using GraphPad Prism software (version 8 for Windows, GraphPad Software, La Jolla California USA, www.graphpad.com).

### Data conversion

For the comparison of the in this study measured values with common used limit values or guidelines, the data of NO and NO_2_ were converted in µg/m^3^ using the formula^31^:

$$c\left[ {\upmu {\text{g}}/{\text{m}}^{3} } \right] = 0.0409 \times c\ [{\text{ppb}}] \times {\text{MW}}\left[ {{\text{g}}/{\text{mol}}} \right]\left( {{\text{at}}\;1013.25\;{\text{mbar}}\;{\text{and}}\;25\;^\circ {\text{C}}} \right)$$

*c *concentration, *ppb *parts per billion, *MW *molecular weight, *MW NO* = 30.01 g/mol*; MW NO*_*2*_ = 46.01 g/mol.

As the NO_x_ monitor display the data in ppm, all values were multiplied by 1000 to convert to the unit ppb (parts per billion).

## Results

**Figure 1:**
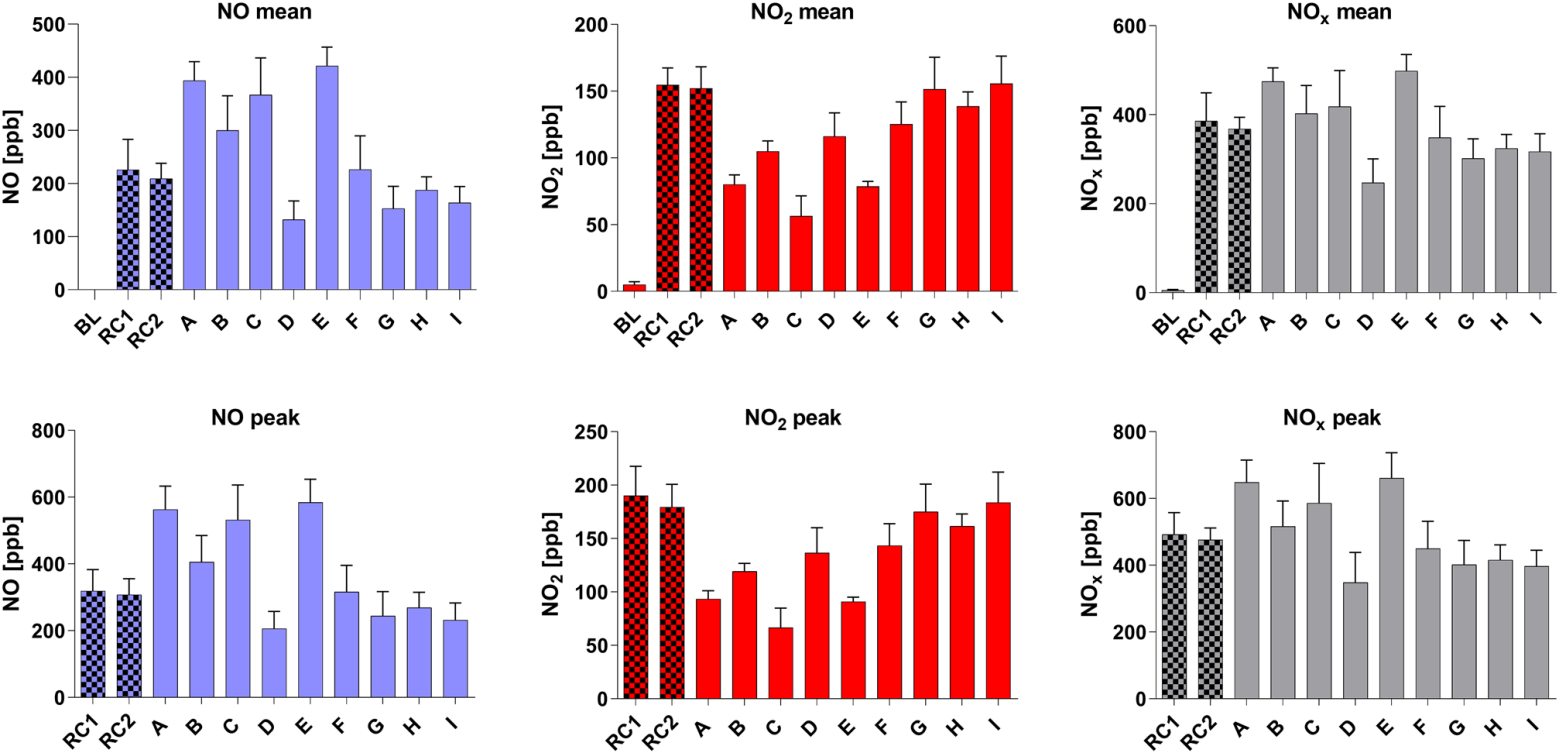
Mean and peak concentrations (ppb) of nitric oxide (NO), nitrogen dioxide (NO_2_), and nitrogen oxides (NO_x_) of the reference cigarette 3R4F (RC1, RC2) and the cigarette brands A to I. Baseline (BL) only given at mean concentrations.

Table 2 and Fig. 1 present the NO, NO_2_, and NO_x_ results of C_mean_ and C_peak_ of all investigated cigarette brands.

**Table 2:** Mean concentrations (C_mean_) and peak concentrations (C_peak_) of nitric oxide (NO), nitrogen dioxide (NO_2_), and nitrogen oxides (NO_x_) of the measured baseline (BL), reference cigarette 3R4F (RC1, RC2), and the cigarette brands A (Marlboro Gold UAE), B (Marlboro Gold GER), C (Winston Red UAE), D (Winston Classic GER), E (Parliament Platinum UAE), F (Parliament Night Blue GER), G (Pall Mall Menthol Blast), H (Winston Menthol), and I (Elixyr Menthol).

	C_mean_ NO (ppb)	C_mean_ NO (µg/m^3^)	C_peak_ NO (ppb)	C_peak_ NO (µg/m^3^)	C_mean_ NO_2_ (ppb)	C_mean_ NO_2_ (µg/m^3^)	C_peak_ NO_2_ (ppb)	C_peak_ NO_2_ (µg/m^3^)	C_mean_ NO_x_ (ppb)	C_peak_ NO_x_ (ppb)
BL	0.11 (0.15)	0.1	n/a	n/a	5.08 (2.14)	9.6	n/a	n/a	5.15 (2.21)	n/a
RC1	226 (57)	277	319 (64)	391	155 (13)	292	190 (28)	357	386 (64)	492 (66)
RC2	209 (29)	256	307 (48)	377	152 (16)	286	180 (21)	339	368 (26)	476 (35)
A	394 (36)	483	563 (70)	691	80 (7)	150	94 (8)	177	475 (30)	648 (66)
B	300 (65)	368	405 (79)	497	105 (8)	197	119 (7)	224	403 (63)	517 (76)
C	367 (70)	450	532 (105)	653	56 (15)	105	67 (18)	126	418 (81)	586 (119)
D	132 (35)	162	207 (51)	254	116 (18)	218	137 (23)	258	247 (54)	349 (90)
E	422 (35)	518	584 (70)	716	79 (4)	149	91 (4)	171	499 (37)	661 (77)
F	227 (63)	278	316 (80)	388	125 (17)	125	144 (20)	271	349 (70)	450 (82)
G	153 (42)	188	245 (72)	301	151 (24)	284	175 (26)	329	302 (44)	402 (73)
H	188 (25)	231	269 (46)	330	139 (11)	261	162 (11)	305	324 (32)	416 (45)
I	164 (30)	201	232 (52)	285	156 (21)	293	184 (28)	346	317 (40)	397 (48)

*UAE *United Arab Emirates, *GER *Germany. The given concentrations in the unit µg/m^3^ were calculated using the formula *c *(µg/m^3^) = 0.0409 × *c *(ppb) ×*MW *(g/mol) (at 1013.25 mbar and 25° C, c = concentration, ppb = parts per billion, MW = molecular weight, MW NO = 30.01 g/mol, MW NO_2_ = 46.01 g/mol). Standard deviations are stated in brackets. n/a = not available.

Regarding NO, the range of the C_mean_ values was from 132 to 422 ppb (equal to 162 µg/m^3^ to 518 µg/m^3^ at 1013.25 mbar and 25 °C). For NO_2_, the C_mean_ values ranged from 56 to 156 ppb (equal to 105 µg/m^3^ to 293 µg/m^3^ at 1013.25 mbar and 25 °C). Looking at the C_mean_ data of NO_x_, the values ranged from 247 to 499 ppb.

The ranges of the ascertained peak values (C_peak_) were as followed: NO: 207 ppb to 584 ppb (equal to 254 µg/m^3^ to 716 µg/m^3^ at 1013.25 mbar and 25 °C). NO_2_: 67 ppb to 190 ppb (equal to 126 µg/m^3^ to 357 µg/m^3^ at 1013.25 mbar and 25 °C). NO_x_: 349 ppb to 661 ppb.

To compare the measured NO_x_ values in SHS with the usually NO_x_ concentration in indoor air at the study location, we took continuous data of 61 h where no investigation was done into account recorded between the two measurement campaigns. The mean of the thus collected data resulted in the baselines for NO = 0.072 ppb (equal to 0.1 µg/m^3^ at 1013.25 mbar and 25 °C), NO_2_ = 5.08 ppb (equal to 9.6 µg/m^3^ at 1013.25 mbar and 25 °C) and NO_x_ = 5.15 ppb.

**Figure 2:**
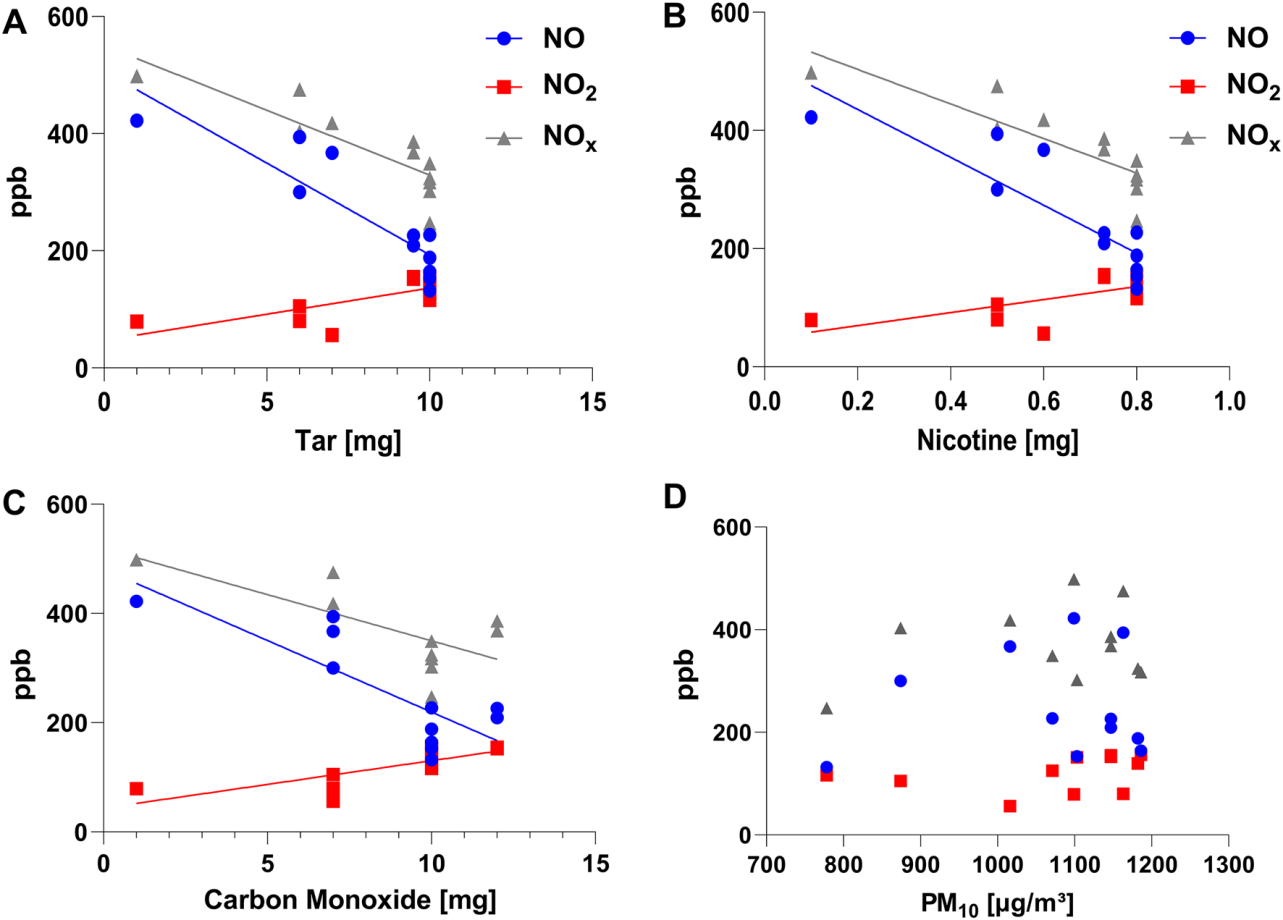
Association between nitric oxide (NO), nitrogen dioxide (NO_2_), and nitrogen oxides (NO_x_) concentrations and concentrations of tar (**A**), nicotine (**B**), carbon monoxide (CO) (**C**), and particulate matter (PM_10_) (**D**). Coefficients of determination (R^2^) of linear regression: NO-tar: 0.779; NO-nicotine: 0.753; NO–CO: 0.684; NO-PM_10_: 0.007; NO_2_-tar: 0.526; NO_2_-nicotine: 0.462; NO_2_-CO: 0.592; NO_2_- PM_10_: 0.105; NO_x_-tar: 0.718; NO_x_-nicotine: 0.724; NO_x_-CO: 0.5; NO_x_-PM_10_: 0.079.

A detailed overview of associations of the C_mean_ values of NO, NO_2_, and NO_x_ with concentrations of tar, nicotine, CO, and PM_10_ shows Table 3. Additionally, Fig. 2 presents the correlations between NO, NO_2_, and NO_x_ and the stated amounts of tar (A), nicotine (B), CO (C), and the measured values of PM_10_ (D). The measured NO data are negatively correlated with the concentrations of tar, nicotine, and CO as specified by the cigarette manufacturers. The concentrations of NO_2_ correlates positively with the stated values of tar, nicotine, and CO. NO_x_ correlated negatively with the concentrations of tar, nicotine, and CO, but in the case of CO without significance. The measured PM_10_ values show no correlations with the concentrations of NO, NO_2_, and NO_x_.

**Table 3:** Spearman correlations of the C_mean_ values of NO, NO_2_, and NO_x_ with concentrations of tar, nicotine, CO (as specified by the cigarette manufacturers), and PM_10_.

	Spearman r	95% Confidence Interval	R squared	*P* value (two-tailed)	*P* value summery	Significant? (*P*< 0.05)
NO versus Tar	− 0.87	− 0.9664 to − 0.543	0.75	0.001	**	Yes
NO versus Nicotine	− 0.87	− 0.9664 to − 0.543	0.75	0.001	**	Yes
NO versus CO	− 0.67	− 0.9098 to − 0.099	0.45	0.027	*	Yes
NO versus PM	0.10	− 0.6743 to 0.5429	0.11	0.760	ns	No
NO_2_ versus Tar	0.62	0.0163 to 0.8943	0.39	0.046	*	Yes
NO_2_ versus Nicotine	0.62	0.0163 to 0.8943	0.39	0.046	*	Yes
NO_2_ versus CO	0.85	0.5075 to 0.9630	0.73	0.001	**	Yes
NO_2_ versus PM	0.57	− 0.0666 to 0.8764	0.32	0.071	ns	No
NO_x_ versus Tar	− 0.93	− 0.9838 to − 0.7521	0.87	< 0.001	***	Yes
NO_x_ versus Nicotine	− 0.93	− 0.9838 to − 0.7521	0.87	< 0.001	***	Yes
NO_x_ versus CO	− 0.60	− 0.8879 to 0.0148	0.36	0.053	ns	No
NO_x_ versus PM	− 0.04	− 0.6378 to 0.5866	< 0.01	0.909	ns	No

*P* values show the significance of the correlations. *ns* = not significant (*P* ≥ 0.05). * = significant (*P* = 0.01 to 0.05). ** = very significant (*P* = 0.001 to 0.01). *** = very significant (*P* < 0.001).

## Discussion

The measured indoor baseline concentration revealed for NO_2_ a mean value of 9.6 µg/m^3^ (5.08 ppb). That is in line with previous studies on NO_2_ indoor concentrations^20^. Our findings show remarkable rises of NO_x_ in small indoors by the smoke of only one cigarette. Of all ten tested cigarette brands, the measured C_mean_ values of five brands exceeded the WHO one-hour mean guideline of 200 µg/m^3^ for NO_2_^6^ by 9% to 47%. The remaining five brands showed between 1.5% and 47% lower NO_2_ values compared to the WHO guideline. The measured NO_2_ C_mean_ value of all examined cigarettes was 215 µg/m^3^ (119 ppb) and, therefore, exceeded the WHO guideline by 8%. Regarding the detected C_peak_ values of NO_2_, six brands exceeded the WHO guideline even in a range from 12 to 79%. Four brands were 12% to 37% below the guideline. The NO_2_ C_peak_ mean value of all cigarettes was 264 µg/m^3^ (140 ppb) and consequently 32% higher than the WHO guideline value. The WHO state unambiguously “that NO_2_—at short-term concentrations exceeding 200 µg/m^3^—is a toxic gas with significant health effects”^6^. It should be borne in mind that the WHO annual mean guideline for NO_2_ is with 40 µg/m^3^ even stricter^4^. There are other guidelines often following the WHO indoor and ambient guidelines for NO_2_, but some differ^32^. At Health Canada, for example, there is an indoor short-term limit value of 170 µg/m^3^ and a long-term limit value of 20 µg/m^3^ based on toxicological data^33^.

Several former studies dealt with NO_x_ emitted by tobacco products in normal-sized indoor rooms. Cyrys et al.^20^ reported NO_2_ mean concentrations in living rooms in Hamburg and Erfurt (both Germany) of 17 µg/m^3^ and 15 µg/m^3^, respectively, an increase of 18% in smokers’ homes, and an increase of 41% in households using gas for cooking, the main indoor source of NO_2_. Additionally, the authors found that outdoor sources can influence indoor NO_2_ levels more than indoor sources depending on the location and season of year. However, they differentiated between smoking and non-smoking in the living room (including the use or non-use of gas in the household) and reported on their influence on indoor NO_2_ levels in general. They did not report on the influence of one single combustion event (burning cigarette or gas cooking, e.g.) and how this can boost NO_2_ concentration in the indoor air temporarily. Slightly lower NO_2_ mean concentrations (2–3 weeks averaged) were found in Scottish and Irish homes: 12.8 µg/m^3^ (6.82 ppb) in smokers’ homes and 16.9 µg/m^3^ (9.01 ppb) in households where gas was used for cooking^22^. In a 20 m^2^ room with a volume of 57 m^3^, water pipes were smoked in four-hour sessions, and NO and NO_2_ concentrations were measured^21^. The authors reported for the smoking sessions a NO mean concentration of 100 ppb and a NO_2_ mean concentration of 60 ppb, meaning 123 µg/m^3^ and 113 µg/m^3^, respectively. That indicates that smoking significantly increases NO_x_ concentrations also in normal-sized indoor rooms. Our study found remarkable mean values (up to 518 µg/m^3^ for NO and 293 µg/m^3^ for NO_2_) and peak values (up to 716 µg/m^3^ for NO and 357 µg/m^3^ for NO_2_) caused by smoking of only one cigarette. Admittedly, the measuring chamber with an indoor volume of 2.88 m^3^ corresponds more to vehicle indoor volumes than indoor volumes of living rooms. However, it can be assumed that chain-smoking or simultaneously smoking of several cigarettes (by several smokers) will increase NO_x_ concentrations also in larger rooms in a similar way. Therefore, this should be in focus for future studies.

The present findings show statistically significant correlations between the strength of a cigarette brand (amount of tar, nicotine, and CO as stated by the manufacturers) and the measured data of NO, NO_2_, and NO_x_. Interestingly, the lower the tar, nicotine, and CO values, the higher were the measured NO and NO_x_ levels, while the measured NO_2_ levels correlated positively with the cigarette strength but with lower significance. The higher the combustion temperature, the more NO_x_ will be generated^4^. Also, the content of bound nitrogen in the tobacco product in the form of nitrate or nitrosamine compounds, e.g., could influence the NO_x_ amount in tobacco smoke^34,35^. Possibly, “lighter” cigarettes burn at smoking with higher combustion temperatures or contain more bound nitrogen. Further investigations on more numerous cigarette brands with various strengths should also examine the burning temperature and the nitrogen amount of the tobacco product. It was reported that NO and NO_x_ concentrations in the mainstream smoke of cigarettes correlated positively with their strength^36^, whereby generation of mainstream smoke took place in a smoking machine following ISO machine-smoking conditions with an as short as possible distance to the NO_x_ analyzer^37^. This set-up is rather comparable to a smokers’ NO_x_ exposure inhaling mainstream smoke. In opposite to this, we simulated the situation of a person exposed to SHS near a burning cigarette. Other studies focusing on NO_x_ in mainstream smoke detected mainly NO but almost no NO_2_^38–40^. Among others, it was assumed that the reducing conditions near the glowing zone of the cigarette favor the formation of NO as the lower oxide of nitrogen or that reactive volatile organic compounds (VOCs) in the tobacco smoke react with NO_2_^38^. Some NO_2_ was detected in the mainstream smoke from the initial puff, but not from the following puffs, while NO_2_ was detected continuously in the side-stream smoke^40^. Only when using a Cambridge filter pad between the cigarette and the analyzer NO_2_ was observed for each puff. The on the pad sampled smoke of the previous puffs could have interacted with NO in the smoke forming NO_2_. As the pad also acted as a barrier between the cigarette and the analyzer, the smoke could age, and, consequently, NO_2_ values could increase^40^. That indicates that the more toxic NO_2_ is mainly detectable in side-stream smoke and aged smoke but less in mainstream smoke and during a prompt measurement. Since SHS is mainly composed of side-stream smoke (85%)^41^, the detection of NO_2_ in SHS is plausible. In addition, the smoke generated in this study had time to age.

A strength of the present study was that the used measuring set-up in the test chamber allowed to create and investigate SHS in a reproducible way without the exposition of any person to tobacco smoke. A methodological limitation was the low frequency of only three measurements per cigarette by the NO_x_ monitor used. Therefore, the real peak values of NO, NO_2_, and NO_x_ could not be detected in all investigated cigarettes. That resulted possibly in slightly too low C_mean_ and C_peak_ measurement values.

## Conclusion

In the last years, the discussion about NO_x_ and especially NO_2_ generated by diesel vehicles in urban areas has made massive waves. To name is the so-called Dieselgate scandal commenced in September 2015^42^. The focus was set on ambient NO_x_ formation, certainly, influencing also indoor burden by NO_x_. But, smoking is a not neglecting source of NO_x_ in indoor rooms. The present study provides NO_2_ concentrations in SHS generated by smoking cigarettes in small indoors at levels known to be a health hazard. Keeping in mind that the used test chamber (2.88 m^3^) has a similar volume to vehicle cabins^43^, smoking in cars can lead to a hazardous increase of NO_2_ concentration. This risk multiplies accordingly if more than one cigarette is smoked (e.g., chain smoking), there is more than one smoker in the car, or the car is driven with closed windows without sufficient ventilation. Therefore, health authorities’ measures are useful and required to inform about and caution against NO_x_ exposure by smoking in cars and other indoor rooms.

## Data Availability

Datasets of this study are available from the corresponding author upon request.

## References

[1] WHO. World Health Organization. Air pollution, accessed 27 April 2020. http://origin.who.int/airpollution/en/ (2020).

[2] Brunekreef B, Holgate ST (2002). Air pollution and health. Lancet.

[3] Glarborg P, Miller JA, Ruscic B, Klippenstein SJ (2018). Modeling nitrogen chemistry in combustion. Prog. Energy Combust..

[4] Jarvis, D. .J., Adamkiewicz, G., Heroux, M. E., Rapp, R. & Kelly, F. J. Nitrogen dioxide. in *WHO Guidelines for Indoor Air Quality: Selected Pollutants WHO Guidelines Approved by the Guidelines Review Committee* (2010).

[5] EU. European Union. European Commission. Environment. Air Quality Standards, accessed 07 May 2020. https://ec.europa.eu/environment/air/quality/standards.htm (2019).

[6] WHO. World Health Organization. Air quality guidelines for particulate matter,ozone, nitrogendioxide and sulfur dioxide, accessed 07 May 2020. https://www.who.int/phe/health_topics/outdoorair/outdoorair_aqg/en/ (2005).

[7] Schraufnagel DE (2019). Air pollution and noncommunicable diseases: a review by the forum of international respiratory societies' environmental committee, part 2: air pollution and organ systems. Chest.

[8] Cesaroni G (2013). Long-term exposure to urban air pollution and mortality in a cohort of more than a million adults in Rome. Environ. Health Perspect..

[9] Atkinson RW, Butland BK, Anderson HR, Maynard RL (2018). Long-term concentrations of nitrogen dioxide and mortality: a meta-analysis of cohort studies. Epidemiology.

[10] CDC. Centers for Disease Control and Prevention. U.S. Department of Health & Human Services. The National Institute for Occupational Safety and Health (NIOSH, accessed 09 June 2020). https://www.cdc.gov/niosh/npg/npgd0448.html. (2019).

[11] Miller OI, Celermajer DS, Deanfield JE, Macrae DJ (1994). Guidelines for the safe administration of inhaled nitric oxide. Arch. Dis. Child Fetal. Neonatal. Ed..

[12] Arashidani K (1996). Indoor pollution from heating. Ind. Health.

[13] Dennekamp M (2001). Ultrafine particles and nitrogen oxides generated by gas and electric cooking. Occup. Environ. Med..

[14] Raw GJ, Coward SK, Brown VM, Crump DR (2004). Exposure to air pollutants in English homes. J. Expo. Anal. Environ. Epidemiol..

[15] Willers SM (2006). Gas cooking, kitchen ventilation, and exposure to combustion products. Indoor Air.

[16] Gilbert NL (2006). Housing characteristics and indoor concentrations of nitrogen dioxide and formaldehyde in Quebec City Canada. Environ. Res..

[17] Baxter LK, Clougherty JE, Laden F, Levy JI (2007). Predictors of concentrations of nitrogen dioxide, fine particulate matter, and particle constituents inside of lower socioeconomic status urban homes. J. Expo. Sci. Environ. Epidemiol..

[18] Gillespie-Bennett J (2008). Sources of nitrogen dioxide (NO2) in New Zealand homes: findings from a community randomized controlled trial of heater substitutions. Indoor Air.

[19] Kephart JL (2020). Nitrogen dioxide exposures from biomass cookstoves in the Peruvian Andes. Indoor Air.

[20] Cyrys J, Heinrich J, Richter K, Wolke G, Wichmann HE (2000). Sources and concentrations of indoor nitrogen dioxide in Hamburg (west Germany) and Erfurt (east Germany). Sci. Total Environ..

[21] Fromme H (2009). Indoor air contamination during a waterpipe (narghile) smoking session. Food Chem. Toxicol..

[22] Semple S (2012). Contribution of solid fuel, gas combustion, or tobacco smoke to indoor air pollutant concentrations in Irish and Scottish homes. Indoor Air.

[23] Gerber A, Hofen-Hohloch AV, Schulze J, Groneberg DA (2015). Tobacco smoke particles and indoor air quality (ToPIQ-II): a modified study protocol and first results. J. Occup. Med. Toxicol..

[24] Gerharz J (2018). Particulate matter emissions of different brands of mentholated cigarettes. J. Air Waste Manag. Assoc..

[25] Braun M, Al-Qaysi R, Klingelhofer D, Muller R, Groneberg DA (2020). High particulate matter burden of cigarettes from the united arab emirates and germany: Are there country-specific differences?. Int. J. Environ. Res. Public Health.

[26] UK. University of Kentucky, Kentucky Tobacco Research and Development Center, 3R4F Preliminary Analysis, accessed 11 May 2020. https://ctrp.uky.edu/assets/pdf/webdocs/3R4F%20Preliminary%20Analysis.pdf (2018).

[27] BMEL. Bundesministerium für Ernährung und Landwirtschaft. Tabakzusatzstoffe, accessed 11 May 2020. https://service.bmel.de/tabakerzeugnisse/index2.php?site_key=153 (2011).

[28] HORIBA. Ambient NOx monitor APNA-370 Operation Manual, accessed 13 May 2020. https://www.horiba.com/fileadmin/uploads/Process-Environmental/Documents/Manuals_US/Ambient/APNA-370_Operation_manual_e.pdf (2009).

[29] Schimpf-Ing. Electronic Development, accessed 15 May 2020. http://www.schimpf-ing.no/index_e.html (2020).

[30] EPA. United States Environmental Protection Agency. Particulate Matter (PM) Pollution, accessed 06. May 2021. https://www.epa.gov/pm-pollution (2020).

[31] Boguski, T. K. KSU. Kansas State University. CHSR. Center for Hazardous Substance Research. Environmental Science and Technology Briefs for Citizens, accessed 26 May 2020. https://www.teesing.com/files/source/understanding-units-of-measurement.pdf (2006).

[32] Salonen H, Salthammer T, Morawska L (2019). Human exposure to NO2 in school and office indoor environments. Environ. Int..

[33] Health. Canada. Residential indoor air quality guideline: nitrogen dioxide, accessed 02 July 2020. https://www.canada.ca/en/health-canada/services/publications/healthy-living/residential-indoor-air-quality-guideline-nitrogen-dioxide.html (2015).

[34] Adams JD, Lee SJ, Hoffmann D (1984). Carcinogenic agents in cigarette smoke and the influence of nitrate on their formation. Carcinogenesis.

[35] Lam E, Kelley E, Martin S, Buettner G (2003). Tobacco xenobiotics release nitric oxide. Tob. Induc. Dis..

[36] Counts ME, Hsu FS, Laffoon SW, Dwyer RW, Cox RH (2004). Mainstream smoke constituent yields and predicting relationships from a worldwide market sample of cigarette brands: ISO smoking conditions. Regul. Toxicol. Pharmacol..

[37] ISO. Standard 3308, fourth ed., International Organization for Standardization. Routine analytical cigarette-smoking machine–definitions and standard conditions. (2000).

[38] Norman V, Keith CH (1965). Nitrogen oxides in tobacco smoke. Nature.

[39] Jenkins RA, Gill BE (1980). Determination of oxides of nitrogen (NOx) in cigarette smoke by chemiluminescent analysis. Anal. Chem..

[40] Shorter JH (2006). Measurement of nitrogen dioxide in cigarette smoke using quantum cascade tunable infrared laser differential absorption spectroscopy (TILDAS). Spectrochim. Acta A Mol. Biomol. Spectrosc..

[41] Raupach T, Radon K, Nowak D, Andreas S (2008). Passive smoking–health consequences and effects of exposure prevention. Pneumologie.

[42] Grange SK, Farren NJ, Vaughan AR, Davison J, Carslaw DC (2020). Post-dieselgate: evidence of NOx emission reductions using on-road remote sensing. Environ. Sci. Technol. Lett..

[43] EPA. Environmental Protection Agency. Department of Energy. Fuel Economy Guide, accessed 27 May 2020. https://www.fueleconomy.gov/feg/pdfs/guides/FEG2018.pdf (2018).

